# MXene-Based Photocatalysts for Pharmaceutical Wastewater Remediation and Sustainable Energy Conversion: Mechanisms, Interface Engineering, and Future Perspectives

**DOI:** 10.3390/ma19132895

**Published:** 2026-07-06

**Authors:** Zhizhen Feng, Shanshan Han, Hong Yan, Jiaqi Shi, Yuxin Ma, Tongtong Wang, Xingchang Zhang, Junchao Jia

**Affiliations:** 1 Shaanxi Key Laboratory of Qinling Ecological Security, Bio-Agriculture Institute of Shaanxi, Shaanxi Academy of Sciences, Xi’an 710043, China; fengzz@xab.ac.cn (Z.F.); hanss@xab.ac.cn (S.H.); yanhong@xab.ac.cn (H.Y.); zhangxc@ms.iswc.ac.cn (X.Z.); 2 Enzyme Engineering Research Center of Shaanxi Province, Xi’an 710600, China; 3 Institute of Interdisciplinary and Innovation Research, Xi’an University of Architecture and Technology, Xi’an 710055, China; jiaqis_22@163.com (J.S.); yuxinma2026@163.com (Y.M.); wangtongtong@xauat.edu.cn (T.W.)

**Keywords:** MXenes, photocatalysts, catalysis, pharmaceutical contaminants, heterojunctions, wastewater remediation, sustainable energy production

## Abstract

**Highlights:**

- MXene-based photocatalysts for pharmaceutical wastewater are reviewed.
- Charge transfer in MXene-based heterojunctions is discussed.
- Pharmaceutical degradation and ROS pathways are summarized.
- MXene-assisted H_2_, CO_2_, and N_2_ conversion reactions are examined.
- Scalable synthesis, stability, and ML-guided design are outlined.

**Abstract:**

Pharmaceutical residues in wastewater pose persistent ecological and public health risks, creating an urgent need for efficient and sustainable remediation technologies. MXene-based photocatalysts have attracted growing interest owing to their high electrical conductivity, tunable surface chemistry, abundant active sites, and excellent charge-transfer capability. This review summarizes recent advances in MXene-based photocatalytic systems for pharmaceutical wastewater treatment and renewable energy production. Key topics include pharmaceutical degradation pathways, reactive oxygen species generation, ecotoxicological implications, and the multifunctional roles of MXenes as conductive supports, electron mediators, and cocatalysts. Interfacial engineering strategies, including Z-scheme, S-scheme, and Schottky heterojunctions, are discussed with respect to light absorption, charge separation, and interfacial redox reactions. Practical considerations, such as reactor design, life cycle assessment, and techno-economic feasibility, are also addressed. Finally, current challenges and future directions are highlighted, particularly scalable fluorine-free synthesis, improved oxidative stability, and machine learning-assisted material design. This review provides a concise framework for developing stable, efficient, and scalable MXene-based photocatalytic platforms for pharmaceutical wastewater remediation and sustainable energy generation.

## 1. Introduction

Pharmaceutical residues, such as antibiotics, anti-inflammatory drugs, and anticonvulsants, have emerged as contaminants of growing concern because of their continuous release, bioactive nature, persistence, and potential risks to aquatic ecosystems and human health [1,2,3,4]. These compounds are frequently detected in surface water, groundwater, and even drinking water, typically at trace concentrations ranging from 1 ng L^−1^ to 600 ng L^−1^ [5,6,7,8,9]. Despite their low concentrations, many pharmaceuticals are poorly biodegradable and may induce chronic ecological effects, endocrine disruption, and the dissemination of antibiotic-resistant bacteria and antibiotic resistance genes [10,11,12]. Conventional wastewater treatment processes are often inadequate for removing these recalcitrant micropollutants because of limited removal efficiency, high energy or chemical inputs, and potential secondary pollution [13,14,15,16,17,18,19,20,21]. Therefore, semiconductor photocatalysis has attracted considerable interest as an advanced oxidation process for pharmaceutical wastewater remediation, as light-driven reactive species can promote the degradation and mineralization of persistent pharmaceutical contaminants under relatively mild conditions [22,23,24,25].

**Figure 1:**
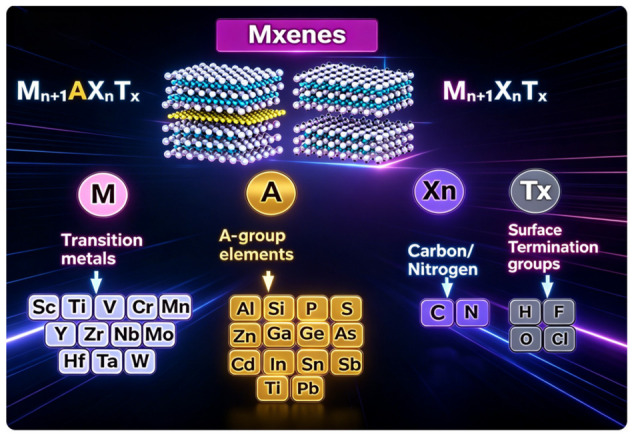
Elemental building blocks of MAX phases and MXenes. Different elements in the periodic table contribute to the formation of MXenes (M_n+1_X_n_T_x_) and MAX phases (M_n+1_AX_n_T_x_). The arrows denote the compositional correspondence between layered structures and their constituent elements. Color coding distinguishes transition metals (M), A-group elements (A), carbon/nitrogen sites (X), and surface terminations (T_x_; –O, –F, and –OH). The background is schematic and used solely to improve visual contrast.

Since the isolation of graphene, two-dimensional nanomaterials have attracted extensive interest in photocatalysis owing to their unique electronic structures, large specific surface areas, and shortened charge-migration pathways [26,27,28]. Among them, MXenes have emerged as an important class of two-dimensional transition metal carbides, nitrides, and carbonitrides since their discovery in 2011 [29,30,31]. MXenes are generally described by the formula M_n+1_X_n_T_x_, where M denotes an early transition metal, X represents carbon or nitrogen, and T_x_ refers to surface terminations such as –O, –F, and –OH. The value of *n* is commonly 1, 2, or 3, corresponding to representative structures such as M_2_X, M_3_X_2_, and M_4_X_3_. A summary of typical MXene compositions and their corresponding MAX-phase precursors is presented in Figure 1. Benefiting from their distinctive layered structures and rich surface chemistry, MXenes exhibit high electrical conductivity, tunable electronic properties, abundant active sites, and strong interfacial coupling capability. These features make MXenes promising platforms for addressing rapid charge recombination and sluggish surface redox kinetics, two major limitations of conventional semiconductor photocatalysts [32].

**Figure 2:**
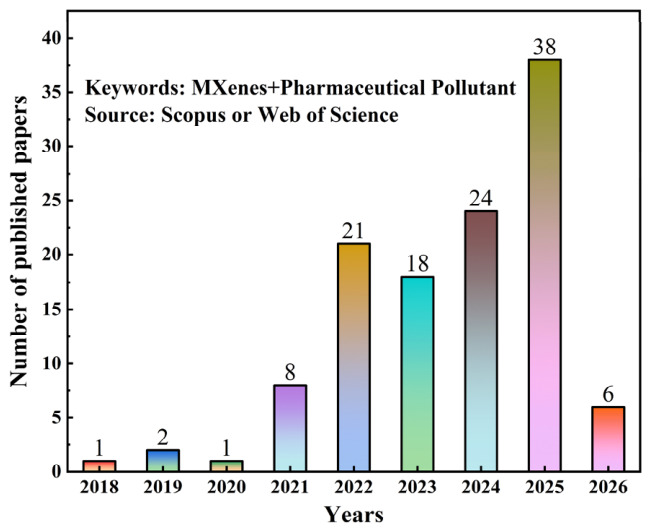
Publication trends in MXene-based photocatalysts for pharmaceutical pollutant degradation. Data were retrieved from Scopus [42]/Web of Science [43] using the keywords “MXene” and “pharmaceutical pollutant degradation” on 29 March 2026.

The metallic conductivity of MXenes can reach approximately 2 × 10^5^ S m^−1^, enabling them to serve as electron acceptors and conductive bridges for promoting interfacial charge separation and suppressing carrier recombination [33,34,35]. Furthermore, hydrophilic surface terminations, including –O, –F, and –OH functional groups, provide abundant active sites for reactant adsorption, semiconductor coupling, and surface redox reactions [36,37,38]. Owing to these structural and physicochemical advantages, MXene-based materials, particularly widely studied Ti_3_C_2_T_x_-based composites, have been increasingly explored for photocatalytic environmental remediation and solar-driven energy conversion, including hydrogen evolution, CO_2_ reduction, and nitrogen fixation [39,40,41]. As illustrated in Figure 2, the rapid growth in related publications reflects the expanding interest in MXene-based photocatalytic systems. This trend also underscores the need for a focused review that links pharmaceutical pollutant degradation with sustainable energy production from the perspective of fundamental charge-transfer mechanisms.

Although MXene-based photocatalysts have been extensively discussed in recent reviews, their potential to bridge pharmaceutical wastewater remediation and renewable energy conversion has not yet been systematically addressed. These two application domains are intrinsically linked by shared charge-transfer processes, interfacial reaction pathways, and photogenerated carrier dynamics. In this review, recent advances in MXene-based photocatalytic systems are critically assessed from a dual-functional perspective. Particular attention is devoted to pharmaceutical degradation pathways, reactive oxygen species generation, and ecotoxicological implications, and the roles of MXenes in solar-driven energy conversion reactions, including hydrogen evolution, CO_2_ reduction, and nitrogen fixation. To elucidate the mechanistic origins of enhanced photocatalytic activity, interfacial engineering strategies are further examined, with emphasis on Z-scheme, S-scheme, and Schottky heterojunctions that regulate light harvesting, charge separation, and directional electron transfer. Finally, key challenges and future research priorities are outlined, including scalable synthesis, oxidative stability, and practical implementation. By integrating mechanistic understanding with application-oriented analysis, this review provides a comprehensive perspective for the rational design and scalable development of next-generation MXene-based photocatalytic technologies.

Furthermore, although several reviews have discussed MXene-based materials for environmental remediation, photocatalysis, membrane separation, or energy conversion, most of them focus on either pollutant removal or solar energy conversion as separate topics. A systematic framework linking pharmaceutical wastewater remediation with sustainable energy production through shared interfacial charge-transfer mechanisms remains insufficiently developed. To clarify the novelty and scope of the present review, representative recent reviews are compared in Table 1.

## 2. MXene-Based Photocatalyst for Pharmaceutical Pollutant Removal

### 2.1. Construction of MXene-Based Heterojunctions

Pristine MXenes possess high electrical conductivity, tunable surface terminations, and abundant active sites. However, they are more commonly employed as cocatalysts, conductive supports, or charge mediators rather than independent photocatalysts [37]. Conventional semiconductors, such as TiO_2_, g-C_3_N_4_, and CdS, often suffer from rapid charge recombination and insufficient visible-light response, which restrict their degradation efficiency toward pharmaceutical pollutants. Therefore, MXenes are frequently coupled with semiconductor photocatalysts to construct heterojunction systems [47].

Efficient interfacial contact is usually established through mechanical self-assembly, ultrasonic dispersion, or in situ hydrothermal growth. Typical examples include CuFe_2_O_4_/Ti_3_C_2_ [48] and MoS_2_/Ti_3_C_2_ [49] composites prepared by hydrothermal methods. In these heterostructures, MXenes can act as electron sinks or conductive bridges, promoting interfacial charge migration and suppressing electron–hole recombination. Depending on the band alignment and interfacial configuration, Schottky junctions, Z-scheme systems, or S-scheme heterojunctions can be formed. These engineered interfaces enhance visible-light response and improve the generation of reactive oxygen species, thereby promoting the degradation and mineralization of pharmaceutical pollutants [37,47].

Surface terminations are central to the electronic behavior of MXenes and strongly influence heterojunction formation. Oxygen-, hydroxyl-, and fluorine-terminated MXenes exhibit different work functions, surface dipoles, hydrophilicity, and interfacial binding strengths [1]. Oxygen-rich terminations often increase the work function of Ti_3_C_2_T_x_ and can strengthen electronic coupling with oxide or sulfide semiconductors, facilitating electron extraction and interfacial charge separation [37]. Hydroxyl groups improve hydrophilicity and pollutant adsorption but may also influence surface protonation and pH-dependent charge transfer. In contrast, excessive –F terminations may reduce hydrophilicity, weaken interfacial coupling, and lower the density of reactive surface sites. Therefore, controlling the type and ratio of surface terminations is essential for optimizing band alignment, Schottky barrier formation, built-in electric fields, and photocatalytic efficiency [50,51,52].

### 2.2. Photocatalytic Degradation Performance Toward Pharmaceutical Compounds

Pharmaceutical compounds, including tetracycline, ciprofloxacin, and diclofenac, are persistent micropollutants that are difficult to eliminate through conventional biological treatment processes [41]. MXene-based heterostructures have demonstrated promising photocatalytic activity toward these contaminants, as summarized in Table 2.

**Figure 3:**
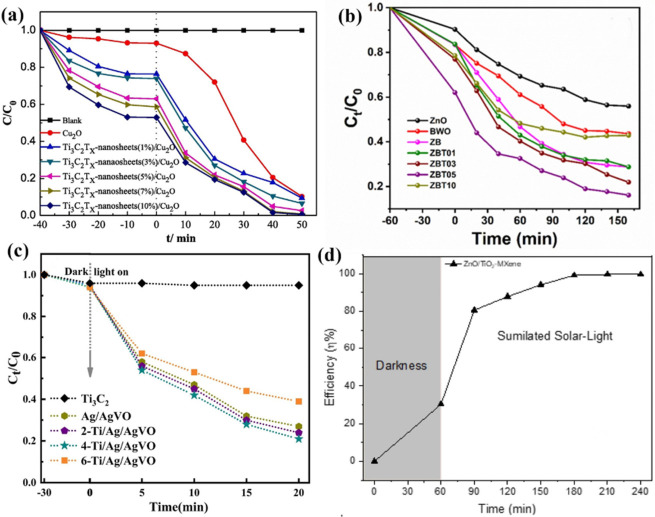
Photocatalytic degradation of pharmaceutical contaminants using MXene-based semiconductor heterostructure photocatalysts. (**a**) Tetracycline degradation curves using different photocatalysts. Reproduced from Ref. [54] with permission from Elsevier; (**b**) Ciprofloxacin degradation performance of ZnO, BWO, and ZB photocatalysts under light irradiation. Reproduced from Ref. [57] with permission from Elsevier; (**c**) Effect of Ti_3_C_2_ content on the photocatalytic degradation efficiency of Ti_3_C_2_/Ag/AgVO composites. Reproduced from Ref. [59] with permission from Elsevier; (**d**) Photodegradation efficiency of ZnO–TiO_2_–MXene photocatalyst toward carbamazepine removal. Reproduced from Ref. [60] with permission from Elsevier.

Recent studies have shown that structural engineering plays a crucial role in enhancing the photodegradation performance of MXene-based composites. To improve catalyst recoverability, Jang et al. introduced magnetic Fe_3_O_4_ into Ti_3_C_2_T_x_, achieving stable diclofenac removal over repeated cycles [53]. Binary heterojunctions are also effective in promoting charge separation; for example, Zhao et al. reported that Ti_3_C_2_T_x_/Cu_2_O degraded 97.6% of tetracycline within 50 min under visible light (Figure 3a) [54]. More complex ternary systems further enhance photocatalytic activity by improving interfacial charge transfer and radical generation [55,56]. Sharma et al. fabricated ZnO/Bi_2_WO_6_/Ti_3_C_2_ for ciprofloxacin removal under sunlight (Figure 3b) [57,58], while Fang et al. developed a Yb^3+^/Tm^3+^ co-doped Ti_3_C_2_/Ag/Ag_3_VO_4_ composite with broadened light absorption (Figure 3c) [59]. In addition, ZnO-TiO_2_-MXene heterostructures showed nearly complete carbamazepine removal under solar irradiation (Figure 3d) [60]. These examples indicate that magnetic separation, heterojunction construction, and broad-spectrum light harvesting are effective strategies for improving pharmaceutical degradation over MXene-based photocatalysts.

The degradation efficiencies listed in Table 2 should not be directly interpreted as an absolute ranking of photocatalytic activity because the reported values were obtained under different initial pollutant concentrations, catalyst dosages, irradiation intensities, pH values, dissolved oxygen levels, and water matrices. In many studies, light intensity and apparent quantum efficiency were not reported, and mineralization efficiency was evaluated less frequently than parent-compound removal. Therefore, when available, apparent rate constants, normalized rate constants, TOC removal, and cycling stability are more informative than single-point degradation percentages. Future studies should report standardized parameters, including photon flux, catalyst mass, illuminated area, pollutant concentration, TOC removal, and catalyst recovery efficiency, to improve reproducibility and cross-study comparability.

**Table 2:** Performance comparison of MXene-based photocatalysts categorized by heterojunction configurations for pharmaceutical wastewater treatment.

Heterojunction Types	Catalyst	Synthesis Method	Pollutant	Key Requirements	Efficiency (%)	TOC Removal (%)	Enhancement Induced by MXene	References
Schottky-like MXene/semiconductor heterojunction	CuFe_2_O_4_/MXene	Simple-sol hydrothermal method	Sulfamethazine	Visible light	59.4%.	~52% after 1 h visible-light irradiation	Electron reservoir/shuttle; enhanced carrier separation	[48]
Magnetic composite	Magnetic-Ti_3_C_2_T_x_	Chemical co-precipitation	Diclofenac	20 W Hg lamp	100% in 30 min	-	MXene provides magnetic recoverability and conductive support for enhanced oxidation	[53]
Schottky heterojunction	Ti_3_C_2_T_x_/Cu_2_O	Precipitation method	Tetracycline	500 W Xenon lamp	97.6% in 50 min	-	Schottky barrier; electron transfer from Cu_2_O to Ti_3_C_2_T_x_	[54]
Type-II Ternary heterojunction	ZnO/Bi_2_WO_6_/Ti_3_C_2_	Electrostatic self-assembly route	Ciprofloxacin	Natural sunlight	77% in 160 min	-	Electron mediator; improved charge separation	[57]
2D/2D van der Waals heterojunction	Mo_2_C/g-C_3_N_4_	Electrostatic self-assembly method	Tetracycline	300 W of Xe lamp	97%	76% within 2 h visible-light irradiation	Electron trap; internal electric field; prolonged carrier lifetime	[58]
Schottky/Z-scheme heterojunction	TiO_2_@Ti_3_C_2_ MXene/Bi_2_S_3_	Hydrothermal method	Tetracycline	150 W Xenon lamp	84.13% in 135 min	-	Schottky barrier; charge mediator; suppressed recombination	[61]
Type-II heterojunction	g-C_3_N_4_/Ti_3_C_2_	Evaporation-induced self-assembly method	Ciprofloxacin	500 W Xenon lamp	100 in 150 min	-	MXene as electron acceptor; Ti_3_C_2_ accepts electrons from g-C_3_N_4_, promoting charge separation	[62]
Ternary heterojunction	g-C_3_N_4_/Ti_3_C_2_/TiO_2_	Electrochemical anodization method	Tetracycline hydrochloride	300 W Xenon lamp	85.12% in 180 min	-	Charge-transfer bridge; inhibited electron–hole recombination	[63]
Ternary heterojunction	Ti_3_C_2_-Bi/BiOCl	Solvothermal method	Ciprofloxacin	300 W Xenon lamp	89% in 100 min	-	Electron trap/shuttle; broadened visible-light absorption	[64]
Ternary heterojunction	CdS/Nb_2_O_5_/Nb_2_C MXene	Hydrothermal	Carbamazepine	Visible light	92%	-	Conductive substrate; promotes charge separation	[65]
Schottky heterojunction	C_3_N_4_/Ti_3_C_2_-MXene	Pre-polymerization and solid mixture-calcination method	Ciprofloxacin	Visible light	99%	37% (120 min, visible light)	Electron mediator; Z-scheme charge transfer	[66]
-	Ti_3_C_2_T_x_	Etching method	Carbamazepine	Solar light	~60%	-	-	[67]
Z-scheme heterojunction	MXene Ti_3_C_2_ derived TiO_2_/g-C_3_N_4_	In situ calcination	Tetracycline	300 W Xenon lamp	TC = (80 min) 83.5%	TC: 66.3% (80 min, visible light)	Electron mediator; enhanced charge separation	[68]
			Ciprofloxacin		CIP = (60 min) 61.7%	CIP: 41.8% (60 min, visible light)		
Type-II heterojunction	CeO_2_/Ti_3_C_2_-MXene	Hydrothermal	Tetracycline	350 W Xenon lamp	80.2% in 60 min	-	Electron acceptor; promotes charge separation	[69]
Z-scheme heterojunction	g-C_3_N_4_/Ti_3_C_2_ MXene/black phosphorous	Calcination method	Ciprofloxacin	300 W Xenon lamp	>99% in 60 min	37% (120 min, visible light)	Electron mediator; Z-scheme charge transfer	[70]
Schottky-like heterojunction	MXene-Ti_3_C_2_/MoS_2_	Hydrothermal	Ranitidine	UV-free 25 W LED lamp	88.4% in 60 min	73.58% (60 min, visible light)	Hole reservoir; suppresses charge recombination	[71]
-	Zero-valent iron (nZVI) @Ti_3_C_2_-MXene	in situ reductive deposition method	Ranitidine	Catalytic degradation	91.1% in 30 min	-	-	[72]
Z-scheme heterojunction	g-C_3_N_4_/Ti_3_C_2_ MXene/MoSe_2_	Calcination method	Enoxacin	300 W Xenon lamp	100% in 60 min	-	Electron mediator; Schottky junction; charge separation	[73]
-	Ti_2_C/TiO_2_ modified with Ag_2_O, Ag, PdO, Pd & Au	Ti_2_C mixed with nanoparticle precursors	Salicylic acid	150 W Hg lamp	95.8% in 200 min	-	Conductive support; electron trapping	[74]
Surface heterojunction (TiO_2_ facets)	Fe modified Ti_3_C_2_	Solvothermal	Carbamazepine	300 W Xenon lamp	100% in 60 min	-	Electron reservoir; Schottky barrier; charge separation	[75]
Schottky heterojunction	Ag_2_WO_4_/Ti_3_C_2_	Electrostatic interactions	Tetracycline hydrochloride	300 W Xenon lamp	62.9% in 40 min	-	Electron acceptor; Schottky junction; charge separation	[76]
			Sulfadimidine		88.6% in 40 min			
2D/2D heterojunction	Bi_2_WO_6_/Ti_3_C_2_	Hydrothermal	Amoxicillin	300 W Xenon lamp	100% in 40 min.	-	Electron mediator; enhanced charge transfer; photothermal effect	[77]
Schottky heterojunction	MXene/Ag_2_S	Chemical deposition and electrostatic self-assembly	Tetracycline hydrochloride	300 W Xenon lamp	94.91% in 75 min	-	Schottky barrier; electron trap; suppressed recombination	[78]
Schottky heterojunction	Ti_3_C_2_/Bi_2_O_2_CO_3_	Hydrothermal	Tetracycline	300 W Xenon lamp	80% in 120 min	-	Conductive support; charge separation	[79]
Dual heterojunction (Type-II + Schottky)	Ti_3_C_2_-MIL-125-NH_2_	Hydrothermal method	Tetracycline hydrochloride	300 W Xenon lamp	82.8% in 60 min	-	Dual heterojunction; enhanced carrier density; charge separation	[80]
Schottky heterojunction	Ti_3_C_2_T_x_/alkalizedg-C_3_N_4_	Simple in situ calcination	Tetracycline hydrochloride	300 W Xenon lamp	77% in 60 min.	-	Electron acceptor; Schottky junction; charge separation	[81]
LDH Composite Heterojunction	Zn/Ti-LDH-MXenes	Hydrothermal method	Acetaminophen	300 W Xe lamp	100% in 40 min	-	Electron mediator; enhanced charge separation	[82]
			Ibuprofen		99.7% in 60 min			
Schottky heterojunction	Ti_3_C_2_ MXene/g-C_3_N_4_ (TiC/SCN)	Ultrasonication method	Arbidol hydrochloride	300 W Xe lamp	99% in 150 min	37.9% (150 min, visible light)	Electron trap; Schottky barrier; charge separation	[83]
Z-scheme heterojunction	g-C_3_N_4_/CATC	One-step H_2_O_2_ oxidation method	Tetracycline	270 W xenon lamp	86.34% in 60 min	-	Direct Z-scheme; enhanced redox capacity; charge separation	[84]
Z-scheme heterojunction	In_2_S_3_/MQDs/SmFeO_3_	Ultrasonication method	Sulfamethoxazole	300 W Xe lamp	98%	-	MQDs as electron mediator; Z-scheme charge transfer	[85]
			4-chlorophenol		95.4%			
S-scheme/Schottky dual heterojunction	BiOBr/Bi_2_MoO_6_/Ti_3_C_2_/MMT_ex_	Microwave-assisted solvothermal method	Levofloxacin	500 W Xenon lamp	99% in 120 min	77% (120 min, visible light)	Schottky/S-scheme; multiple electron transfer pathways; charge separation	[86]
Schottky heterojunction	CaIn_3_S_4_/Ti_3_C_2_T_x_	Hydrothermal method	Tetracycline hydrochloride	500 W Xe lamp	92% in 150 min	-	Schottky junction; electron reservoir; charge separation	[87]

“-” indicates the default.

### 2.3. Structure–Activity Relationships and Performance-Determining Factors of MXene-Based Photocatalysts

The superior performance of some MXene-based photocatalysts over others cannot be attributed solely to the presence of MXenes. Instead, it is governed by a combination of MXene composition, surface terminations, defect density, semiconductor coupling mode, interfacial contact quality, and reaction conditions [35,36,46]. For example, Ti_3_C_2_T_x_ is frequently used because of its high electrical conductivity and favorable work function, enabling it to act as an electron sink or charge bridge. However, the actual charge-transfer efficiency depends strongly on the surface terminations. Oxygen-terminated MXenes usually exhibit higher work functions and stronger interfacial electronic coupling, whereas –F-rich surfaces may reduce hydrophilicity and weaken adsorption or charge-transfer processes. Defects and edge sites can provide adsorption and activation sites, but excessive structural disorder may accelerate oxidation and act as recombination centers. Therefore, high degradation efficiencies reported for MXene-based composites should be interpreted in the context of both intrinsic material properties and experimental conditions [46,51,88].

### 2.4. Charge-Transfer Mechanisms in MXene-Based Heterojunctions

In a Schottky heterojunction, metallic or highly conductive MXenes, such as Ti_3_C_2_T_x_, are coupled with a semiconductor whose Fermi level is usually higher than that of the MXene. After intimate contact, electrons migrate from the semiconductor to the MXene until Fermi-level equilibrium is reached, resulting in interfacial band bending and the formation of a Schottky barrier. Under illumination, photogenerated electrons can be rapidly extracted by the MXene, while holes remain in the semiconductor valence band, thereby suppressing electron–hole recombination and improving interfacial reduction reactions. However, Schottky systems mainly enhance charge separation and electron transport; if the accumulated electrons on MXene cannot be efficiently consumed by O_2_, H^+^, CO_2_, or other acceptors, excessive electron trapping may weaken the overall redox utilization. In contrast, Z-scheme heterojunctions are designed to preserve strong redox potentials. In a typical MXene-mediated Z-scheme system, two semiconductors with staggered band positions are connected by MXene as a solid-state electron mediator or conductive bridge [89]. The electrons with weaker reduction ability recombine with holes of weaker oxidation ability through the MXene interface, whereas the electrons in the more negative conduction band and holes in the more positive valence band are retained for reduction and oxidation reactions, respectively. This pathway is particularly beneficial for pharmaceutical degradation because it maintains strong oxidative species such as holes and ·OH while also enabling reductive activation of dissolved oxygen to •O_2_^−^. S-scheme heterojunctions differ from conventional type-II systems by combining a reduction photocatalyst and an oxidation photocatalyst with different Fermi levels. Upon contact, spontaneous electron migration induces an internal electric field and band bending at the interface [90]. Under light irradiation, low-energy electrons and holes recombine across the interface, while high-energy electrons and holes are spatially preserved. Therefore, S-scheme systems simultaneously promote charge separation and retain strong oxidation/reduction capability, overcoming the thermodynamic limitation of traditional type-II heterojunctions. For MXene-based systems, the distinction among these mechanisms depends strongly on MXene work function, surface terminations, semiconductor band edges, and interfacial coupling quality.

To avoid purely descriptive mechanism assignment, the revised manuscript also emphasizes that Schottky, Z-scheme, and S-scheme pathways should be verified using multiple complementary experimental and theoretical techniques rather than degradation efficiency or radical trapping tests alone [36]. Steady-state photoluminescence spectroscopy can provide preliminary information on electron–hole recombination, while time-resolved photoluminescence gives carrier lifetime information and helps determine whether MXene incorporation accelerates charge separation or introduces recombination centers. Transient photocurrent measurements are useful for evaluating the generation and migration of photogenerated carriers under intermittent illumination, whereas electrochemical impedance spectroscopy reveals interfacial charge-transfer resistance. Mott–Schottky analysis is commonly used to estimate flat-band potentials, semiconductor conductivity type, and approximate band-edge positions, which are essential for evaluating whether the proposed Z-scheme or S-scheme band alignment is thermodynamically feasible. Kelvin probe force microscopy measurements provide direct information on work function and surface potential differences, which are particularly important for MXene-based Schottky and S-scheme systems because MXene surface terminations can strongly regulate Fermi-level positions. In situ or light-irradiated X-ray photoelectron spectroscopy can further track binding-energy shifts and provide evidence for interfacial electron migration direction. Electron paramagnetic resonance spectroscopy using spin-trapping agents can identify reactive oxygen species such as ·•OH, •O_2_^−^, ^1^O_2_, while scavenger experiments help determine the dominant active species involved in pharmaceutical degradation. Nevertheless, radical trapping should be considered supportive rather than conclusive, because scavengers may alter adsorption, pH, or reaction pathways. Density functional theory calculations can complement experiments by predicting work functions, differential charge density, band alignment, adsorption energies, and reaction energy barriers, thereby clarifying how MXene terminations and defects influence charge redistribution. Therefore, a reliable mechanistic interpretation should integrate band-structure analysis, work function measurements, photoelectrochemical characterization, radical identification, operando spectroscopy, and theoretical calculations [46]. This combined approach provides a more rigorous basis for distinguishing Schottky electron sink behavior, MXene-mediated Z-scheme charge recombination, and S-scheme internal electric field-driven carrier separation in MXene-based photocatalysts.

### 2.5. Photocatalytic Mechanisms and Degradation Pathways

**Figure 4:**
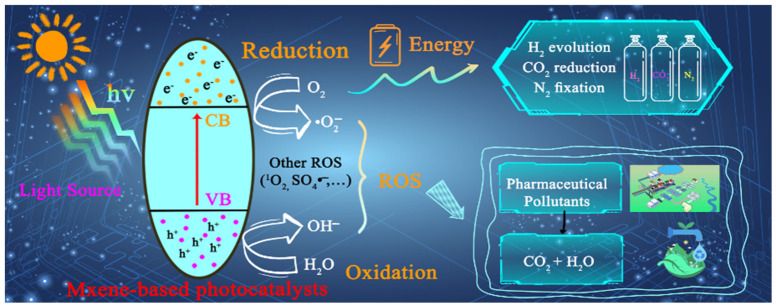
Schematic representation of the photocatalytic mechanism for pharmaceutical pollutant remediation. The arrows indicate light-induced charge transfer, ROS generation, and pollutant degradation pathways, while the colors and lines are used to distinguish different reaction species and schematic functional regions without representing quantitative differences.

Pharmaceutical photodegradation is generally initiated by photoinduced charge generation and governed by the subsequent formation of reactive oxygen species (ROS). Upon light excitation, electrons are transferred from the valence band (VB) to the conduction band (CB), leaving holes in the VB [91]. In MXene-based composites, the conductive MXene component acts as an interfacial charge mediator, promoting charge separation, suppressing recombination, and enhancing ROS production. These processes collectively drive the oxidative degradation and mineralization of pharmaceutical contaminants. The overall mechanism is illustrated in Figure 4, and the key ROS-generation reactions are summarized in Equations (1)–(6).

Photocatalyst + hυ → h^+^_VB_ + e^−^_CB_ (1)

e^−^_CB_ + O_2_ → •O_2_^−^ (2)

•O_2_^−^ + H^+^→ •OOH (3)

H_2_O_2_ + e^−^_CB_ → •OH + OH^−^ (4)

H_2_O/OH^−^ + h^+^_VB_ → •OH + H^+^ (5)

ROS (•O_2_^−^, •OH) + h^+^_VB_ + Pharmaceuticals → Intermediates → CO_2_ + H_2_O (6)

Elucidating degradation pathways is crucial for understanding the structural evolution and detoxification of pharmaceutical pollutants. For tetracycline, Jin et al. showed that ROS-mediated C–C and C–N bond cleavage over BP/CN generated a series of intermediates before complete mineralization into CO_2_ and H_2_O [92]. For ciprofloxacin, Ahmad et al. identified three major transformation routes over MnO/g-C_3_N_4_, including piperazine ring oxidation, quinolone moiety cleavage, and defluorination/decarboxylation, ultimately producing small inorganic species such as CO_2_, H_2_O, NO_3_^−^, and F^−^ [93].

The efficiency of these oxidative pathways is largely dictated by interfacial charge-transfer kinetics. A 2D/2D Mo_2_C/g-C_3_N_4_ van der Waals heterojunction was shown to establish an internal electric field via Fermi-level equilibration, enabling efficient spatial separation of photogenerated carriers [58]. In TiO_2_@Ti_3_C_2_/Bi_2_S_3_, the conductive Ti_3_C_2_ phase functioned as an electron mediator in an MXene-bridged Z-scheme pathway, preserving strong redox potentials while suppressing recombination [61]. These interfacial effects favor the generation of •O_2_^−^ and •OH, which drive the oxidative degradation and mineralization of tetracycline.

### 2.6. Photocatalytic Performance and Anti-Interference Mechanisms in Real Wastewater

**Figure 5:**
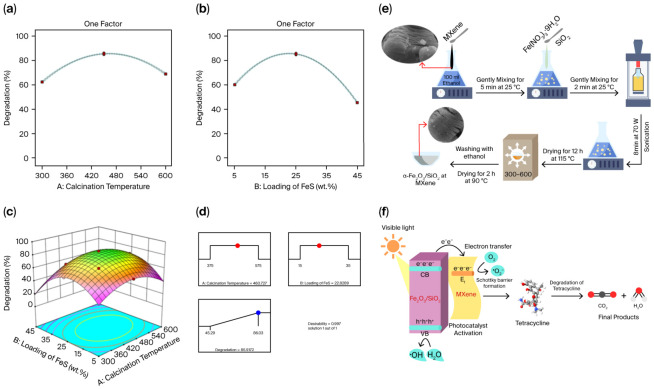
Photocatalytic antibiotic degradation over MXene-based composite photocatalysts. (**a**) Effect of calcination temperature on antibiotic degradation efficiency. (**b**) Effect of FeS loading on antibiotic degradation efficiency. (**c**) Response-surface analysis of the combined effects of calcination temperature and FeS loading. (**d**) Desirability plots for optimizing the reaction variables and degradation response. (**e**) Schematic synthesis of the FeS/MXene photocatalyst. (**f**) Proposed visible-light-driven charge-transfer and tetracycline degradation mechanism over 25FeS/MX-450. Arrows denote the synthesis sequence, charge migration, ROS formation and degradation pathways. Colours distinguish catalyst components, charge carriers, reactive species, pollutants and degradation products. Reproduced from Ref. [94] with permission from Springer Nature.

Although MXene-based photocatalysts have shown promising activity toward pharmaceutical degradation, most evaluations are still performed in simplified single-pollutant systems using deionized water [44]. In contrast, real pharmaceutical wastewater contains complex inorganic ions and natural organic matter, which can profoundly reshape photocatalytic kinetics by competing for reactive oxygen species, photons, and surface-active sites [45,94]. Inorganic anions such as Cl^−^, SO_4_^2−^, and HCO_3_^−^/CO_3_^2−^ can scavenge photogenerated holes or •OH, generating less oxidative secondary radicals, including chlorine- and carbonate-derived radicals (Figure 5) [95,96]. Meanwhile, humic and fulvic acids inhibit photocatalysis through light-screening effects and surface fouling, where adsorption onto MXene-based catalysts blocks active sites and perturbs interfacial charge transfer [46,50]. These matrix effects collectively suppress ROS utilization and reduce the mineralization efficiency of recalcitrant pharmaceutical pollutants.

MXenes possess rich interfacial chemistry arising from their surface terminations, mainly –OH, –O, and –F groups, and high hydrophilicity [51]. While these features favor pollutant enrichment and charge transfer, they also render MXene surfaces susceptible to competitive adsorption and fouling in complex wastewater matrices. Recent efforts have therefore emphasized antifouling engineering and selective adsorption regulation to enhance matrix tolerance [97]. Hydrophobic polymer grafting or steric hindrance design can mitigate natural organic matter adsorption while preserving the diffusion of small pharmaceutical molecules [97]. In addition, magnetic MXene/g-C_3_N_4_/Fe_3_O_4_ composites retained over 90% degradation efficiency for azithromycin and clindamycin in real hospital wastewater and enabled rapid catalyst recovery from sludge-containing systems [98,99]. Accordingly, future assessment should shift from apparent removal efficiency toward multidimensional performance metrics, including selectivity, anti-passivation capability, matrix tolerance, and recyclability under realistic wastewater conditions.

The transition from synthetic solutions to real pharmaceutical wastewater remains a major bottleneck for MXene-based photocatalysis. In real hospital or pharmaceutical effluents, pharmaceutical residues coexist with inorganic ions, natural organic matter, proteins, surfactants, suspended solids, and disinfectant residues [24,98]. These constituents can compete with target pollutants for active sites and reactive oxygen species, attenuate light penetration, and induce surface fouling or passivation of MXene-based catalysts [98]. Therefore, high degradation efficiency in deionized water does not necessarily guarantee comparable performance in real wastewater. Future studies should evaluate MXene-based photocatalysts using real effluents, mixed pharmaceutical systems, continuous-flow reactors, and long-term operation tests [100,101]. In addition to parent-compound removal, TOC removal, toxicity evolution, catalyst leaching, and post-treatment water quality should be systematically evaluated and reported.

### 2.7. Ecotoxicological Assessment of Photocatalytic Degradation Intermediates

The ultimate goal of photocatalytic treatment for pharmaceutical wastewater is not only to decrease the concentration of target contaminants but also to reduce the ecological risks associated with the treated effluent [102]. Although MXene-based photocatalysts have frequently shown high degradation efficiencies toward pharmaceuticals, the environmental safety of the transformation process remains insufficiently evaluated. Structurally complex molecules, such as tetracycline and ciprofloxacin, may undergo incomplete oxidation reactions during photocatalysis, including hydroxylation, demethylation, ring opening, and side-chain cleavage, thereby generating various transformation products [44]. The resulting intermediates can exhibit toxicological profiles that differ from, or even exceed, those of the parent compounds. Thus, LC–MS-based pathway identification alone cannot confirm detoxification, and ecotoxicological assessment should be integrated into degradation analysis.

The electronic conductivity and surface chemistry of MXenes facilitate interfacial charge transfer in Schottky, Z-scheme, and S-scheme heterojunctions. By regulating charge migration and separation, these structures affect the formation of reactive oxygen species (ROS), such as •OH, •O_2_^−^, and photogenerated holes [103,104]. Since these oxidative species differ in redox potential and reaction selectivity, the dominant ROS pathway can influence both the degradation route and the distribution of transformation products. In general, •OH-dominated oxidation is more likely to promote non-selective attack, aromatic ring cleavage, and deep oxidation, which may favor toxicity attenuation. In contrast, •O_2_^−^ dominated pathways may induce partial oxidation or side-chain modification and lead to transient toxic intermediates. Nevertheless, ROS–toxicity relationships are system-dependent and should not be inferred solely from radical trapping results or proposed pathways.

**Figure 6:**
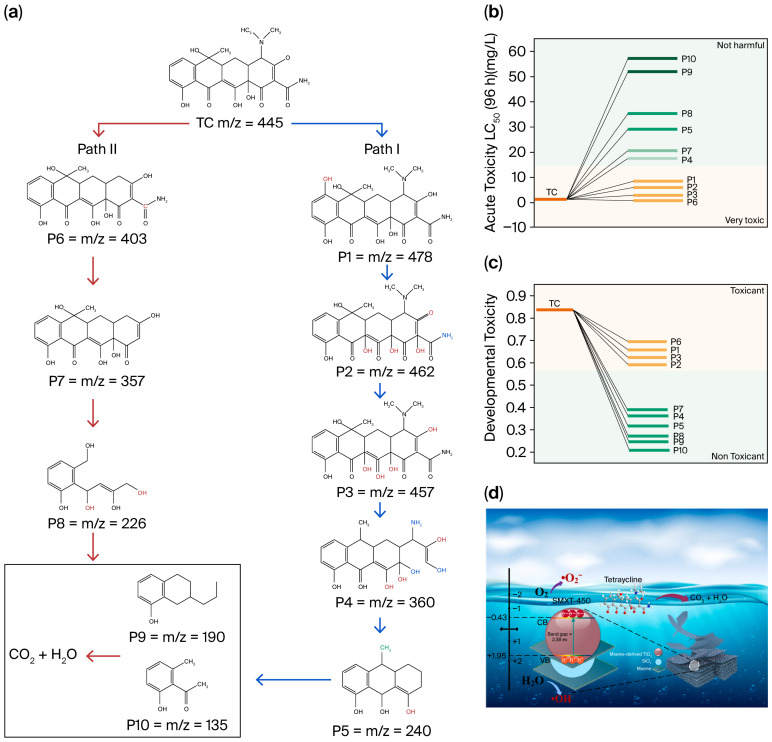
Degradation pathway, toxicity evolution, and photocatalytic mechanism of TCH degradation over SMXT-450. (**a**) Proposed degradation pathways of tetracycline hydrochloride (TCH); (**b**,**c**) Predicted acute and developmental toxicity of TCH and its transformation intermediates, evaluated using the T.E.S.T. consensus method; (**d**) Proposed photocatalytic reaction mechanism for TCH degradation over SMXT-450. Reproduced from Ref. [105] with permission from Elsevier.

Computational toxicology complements intermediate identification by predicting the toxicity of parent compounds and transformation products. Tools such as the U.S. EPA ECOSAR model and the T.E.S.T. consensus method are commonly used to estimate acute and chronic toxicity endpoints of parent compounds and transformation products for representative aquatic organisms, including fathead minnows, Daphnia magna, and green algae, using indicators such as LC_50_, EC_50_, and chronic toxicity values (Figure 6) [105]. These tools are useful for preliminary screening when authentic standards are unavailable, but their reliability is limited by applicability domains, descriptors, and training datasets. Therefore, predictions should be supported by bioassays, such as *Aliivibrio fischeri* luminescence inhibition, *Escherichia coli* growth inhibition, algal growth inhibition, and *Daphnia magna* acute toxicity tests [106]. Integrating molecular identification, toxicity prediction, and biological validation enables a more reliable assessment of whether photocatalysis truly leads to detoxification rather than merely parent-compound disappearance.

### 2.8. Photocatalytic Reactor Design and Scale-Up for Practical Applications

Although MXene-based photocatalysts have shown remarkable activity in slurry systems, translating their laboratory-scale performance into practical wastewater treatment remains challenging from a reactor engineering perspective [100]. Two-dimensional MXene nanosheets readily restack in aqueous media, reducing the accessibility of active sites, while their intrinsically black or dark-gray appearance causes pronounced light-shielding effects [107]. In conventional large-volume suspension reactors, the penetration depth of sunlight or LED irradiation is often limited to several millimeters or a few centimeters, creating extensive dark zones and substantially lowering photon utilization efficiency [101]. In addition, the post-treatment recovery of nanoscale MXene powders from continuous-flow effluents is costly and technically difficult, which may lead to catalyst loss and secondary contamination.

**Figure 7:**
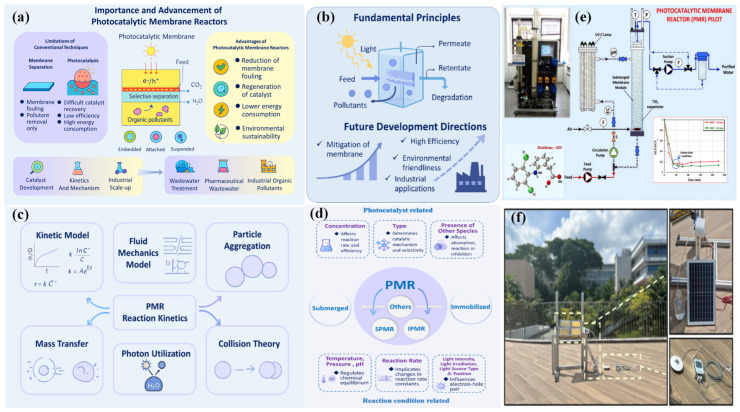
Photocatalytic membrane reactors for wastewater treatment from operating principles to scale-up strategies. (**a**) Synergistic coupling of photocatalysis and membrane filtration in PMRs, highlighting their mechanisms, advantages, and representative applications; (**b**) Operating principles and prospective development trends of PMRs; (**c**) Schematic representation of reaction kinetics in PMR systems; (**d**) Critical factors affecting PMR performance, including photocatalyst properties, operational parameters, and reactor configuration. Reproduced from Ref. [101] with permission from MDPI; (**e**) Pilot-scale continuous photocatalytic membrane reactor. Reproduced from Ref. [110] with permission from Elsevier; (**f**) Digital photograph of a scale-up reactor under ambient conditions. Reproduced from Ref. [111] with permission from Springer Nature. Arrows denote process flow, mass transfer, reaction pathways, charge or species migration, and scale-up connections, while colors distinguish functional modules, reactor components, materials, pollutants, and operating factors.

To address these limitations, photocatalytic membrane reactors, or PMRs, provide a promising engineering platform for immobilizing MXene-based photocatalysts and integrating photocatalytic degradation with membrane separation (Figure 7) [101]. Loading MXene-based composites onto porous supports, such as PVDF or ceramic membranes, by vacuum filtration, electrospinning, or in situ growth can mitigate catalyst recovery issues while enabling cross-flow operation to improve mass transfer and reduce concentration polarization [100]. From a reactor design perspective, microchannel reactors and fixed-bed cross-flow reactors with high specific surface areas are particularly attractive because they can enlarge the illuminated catalytic interface and mitigate the light-shielding limitations of MXenes [108]. During scale-up, rigorous computational fluid dynamics, or CFD, models should be developed to systematically couple and optimize photon-flux distribution, hydrodynamic residence time, and mass-transfer coefficients within the reactor [100,109]. In parallel, scalable synthesis and immobilization routes should be developed to bridge the gap between gram-scale laboratory preparation and kilogram- or ton-scale continuous-flow processing. Such advances will be critical for translating MXene-based photocatalysts from proof-of-concept studies to practical environmental applications.

## 3. Sustainable Energy Production and Additional Applications

The functions that enable MXene-based heterojunctions to enhance photocatalytic pollutant mineralization, including efficient charge separation, suppressed recombination, and rapid interfacial electron transfer, are also central to solar-driven energy conversion. In this context, MXenes can act as conductive electron reservoirs, interfacial charge mediators, and surface chemistry modulators, thereby addressing the sluggish multi-electron kinetics that typically constrain photocatalytic hydrogen evolution, CO_2_ reduction, and nitrogen fixation. Accordingly, this section extends the discussion from environmental remediation to sustainable energy production and other emerging applications, with particular emphasis on how MXene integration regulates charge dynamics, surface reactions, and catalytic selectivity.

### 3.1. Photocatalytic Hydrogen Production

Hydrogen is widely regarded as a clean energy carrier with a high gravimetric energy density of approximately 120 MJ kg^−1^. Photocatalytic water splitting provides a sustainable route for hydrogen production, but its efficiency is often limited by sluggish hydrogen evolution reaction (HER) kinetics and rapid recombination of photogenerated charges [112,113,114,115]. In MXene-based photocatalytic systems, the metallic conductivity of MXenes enables efficient electron extraction and transport, while their tunable surface terminations and abundant interfacial sites can regulate H* adsorption and lower the kinetic barrier for HER [116,117].

**Figure 8:**
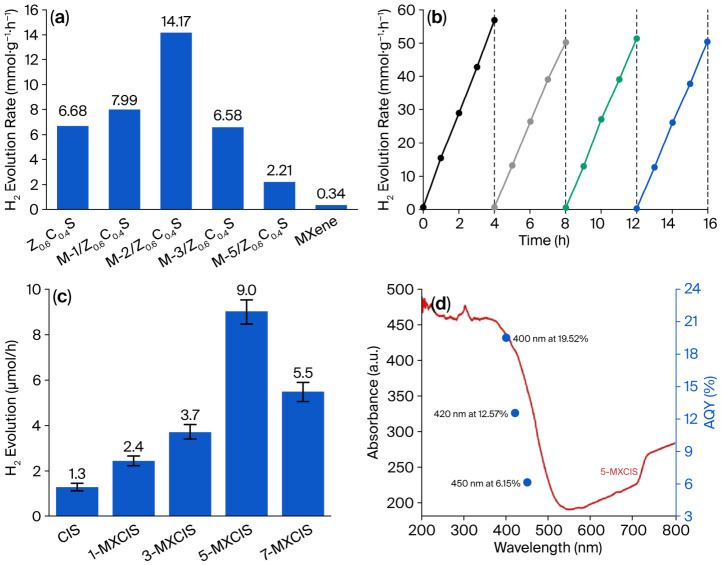
MXene-based composite photocatalysts for solar-driven hydrogen evolution showing activity, stability, and wavelength-dependent quantum efficiency. (**a**) Photocatalytic H_2_ evolution activity of MXene/Z_x_C_1−x_S composites; (**b**) Cycling stability of the optimized M_2_/Z_0.6_C_0.4_S sample during photocatalytic H_2_ evolution. Reproduced from Ref. [88] with permission from Elsevier; (**c**) Visible-light-driven H_2_ generation rates of CIS and x-M_X_CIS photocatalysts; (**d**) Wavelength-dependent apparent quantum yields (AQYs) of 5-M_X_CIS. Reproduced from Ref. [118] with permission from Elsevier.

The rational integration of MXenes with metal sulfides has attracted considerable attention for photocatalytic H_2_ evolution. Metal sulfides generally show strong visible-light absorption and suitable band structures, but their performance is often limited by rapid charge recombination and photocorrosion. MXenes can alleviate these limitations by extracting photogenerated electrons and accelerating interfacial charge transfer. For example, Cao et al. prepared MXene/Zn_x_Cd_1−x_S composites, among which the optimized Z_0.6_C_0.4_S/MXene sample delivered a hydrogen evolution rate of 14.17 mmol g^−1^ h^−1^, 2.1 times that of pristine Zn_x_Cd_1−x_S (Figure 8a). The optimized composite also retained stable activity over four consecutive cycles, suggesting improved photocatalytic stability after MXene incorporation (Figure 8b) [88]. Schottky heterojunctions further highlight the role of MXenes in directional charge migration. Liu et al. investigated Ti_3_C_2_/CdIn_2_S_4_ Schottky heterojunctions, where the optimized 5 wt% Ti_3_C_2_-loaded sample showed the highest H_2_ production rate of 9 μmol h^−1^ (Figure 8c). Its apparent quantum yields reached 19.52%, 12.57%, and 6.15% at 400, 420, and 450 nm, respectively, confirming effective visible-light utilization (Figure 8c,d) [118]. Together, these examples show that MXenes can enhance H_2_ evolution mainly by promoting electron extraction, suppressing charge recombination, and improving interfacial HER kinetics.

### 3.2. Photocatalytic CO_2_ Reduction

The continuous increase in CO_2_ emissions from fossil fuel combustion has intensified the greenhouse effect, making solar-driven CO_2_ conversion into value-added fuels and chemicals, such as CO and CH_4_, an attractive strategy for both carbon mitigation and renewable energy storage [119]. However, photocatalytic CO_2_ reduction is intrinsically challenging because it involves CO_2_ adsorption and activation, multi-electron/proton transfer, product desorption, and competition with the hydrogen evolution reaction. In MXene-based photocatalytic systems, the metallic conductivity of MXenes can facilitate interfacial electron transport, while their tunable surface terminations and abundant active sites can promote CO_2_ chemisorption and stabilize key reaction intermediates. These features make MXenes promising components for improving both the activity and selectivity of photocatalytic CO_2_ reduction.

**Figure 9:**
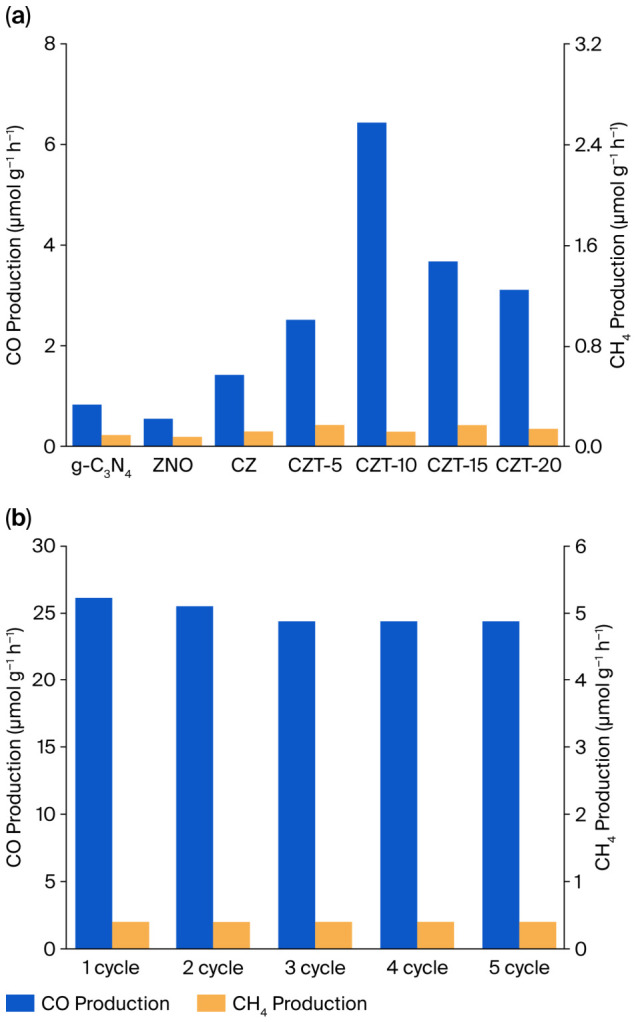
Photocatalytic CO_2_ reduction performance of MXene-based composite photocatalysts. (**a**) CO_2_ reduction activities of representative photocatalyst samples; (**b**) Cycling stability of CZT-10 during photocatalytic CO_2_ reduction. Reproduced from Ref. [120] with permission from Elsevier.

Li et al. investigated Ti_3_C_2_ MXene-modified g-C_3_N_4_/ZnO photocatalysts for CO_2_ reduction [120]. As shown in Figure 9a, the optimized CZT-10 composite achieved a CO production rate of 6.41 μmol g^−1^ h^−1^ and a much lower CH_4_ production rate of 0.26 μmol g^−1^ h^−1^, indicating CO as the dominant reduction product. Cycling tests showed that CO and CH_4_ production remained nearly unchanged over five consecutive cycles, confirming the good stability of CZT-10 (Figure 9b). In addition, the apparent quantum efficiencies for CO and CH_4_ formation at 350 nm were 0.22% and 0.06%, respectively. These results suggest that Ti_3_C_2_ incorporation promotes charge separation and improves selective CO_2_-to-CO conversion in g-C_3_N_4_/ZnO-based photocatalysts [120].

MXene-based Schottky heterojunctions offer an effective strategy for regulating charge migration in CO_2_ photoreduction. Chen et al. prepared TiO_2_/Ti_3_C_2_ composites through the in situ growth of TiO_2_ on Ti_3_C_2_ MXene, forming an intimate Schottky interface [121]. The optimized composite achieved CO and CH_4_ evolution rates 2.8 and 4.0 times higher than those of pristine TiO_2_, respectively, mainly due to accelerated interfacial electron transfer and improved electron–hole separation. These results highlight the potential of Ti_3_C_2_-derived Schottky junctions for enhancing CO_2_ photoreduction.

### 3.3. Photocatalytic Nitrogen Fixation

The strong N≡N triple bond of atmospheric N_2_, with a bond energy of approximately 940.95 kJ mol^−1^, makes nitrogen activation kinetically difficult. Photocatalytic N_2_ fixation under ambient conditions offers a sustainable alternative to the energy-intensive Haber–Bosch process, but it is still limited by inefficient N_2_ adsorption/activation, sluggish six-electron transfer kinetics, and competition from HER [122,123]. In this regard, MXenes have emerged as promising components for photocatalytic nitrogen reduction because their metallic conductivity can facilitate electron extraction and transport, while their abundant surface terminations and defect sites can promote N_2_ chemisorption and activation [124,125,126,127].

To accelerate nitrogen reduction reaction (NRR) kinetics, MXene-based ternary heterojunctions have been developed by integrating conductive MXene frameworks with semiconductor photocatalysts and metal cocatalysts. Hao et al. reported a RuO_2_-loaded TiO_2_–Ti_3_C_2_ photocatalyst for nitrogen fixation, in which Ti_3_C_2_ served as an electron-conducting platform and RuO_2_ acted as a cocatalyst to facilitate N_2_ activation [128]. This ternary structure promoted photogenerated charge separation and transfer, thereby improving photocatalytic nitrogen fixation performance under Xe-lamp irradiation [128].

Transition metal site modulation provides another effective strategy for improving N_2_ adsorption and activation. Gao et al. developed an in situ Co-modified MXene/TiO_2_ heterostructure, in which Co sites modulated the chemisorption behavior of N_2_ and NH_3_, thereby facilitating N_2_ activation and product desorption [129]. The optimized MXene/TiO_2_/Co-0.5% composite achieved an NH_4_^+^ production rate of 110 μmol g^−1^ h^−1^ in pure water without any hole sacrificial agent under UV–vis irradiation, while maintaining good stability under both N_2_ and air atmospheres [129]. These results indicate that transition metal site modulation can enhance photocatalytic nitrogen fixation by improving interfacial charge transfer, strengthening N_2_ activation, and optimizing NH_3_ desorption [129].

As shown in Table 3 and previous research findings, in H_2_ evolution, MXene primarily reduces interfacial electronic transport resistance and improves H• adsorption. In CO_2_ reduction, MXene can enhance CO_2_ adsorption and stabilize intermediates such as •COOH and •CO, but selectivity control remains insufficient; in N_2_ fixation, MXene defects, edge sites, and transition metal modifications aid in N_2_ activation, but NH_3_ contamination, background nitrogen sources, and detection accuracy must be strictly controlled. The common mechanism underlying energy conversion and pollutant degradation is “directed migration of photo-generated charges and regulation of interfacial multi-electron reactions.”

### 3.4. Density Functional Theory Insights into MXene-Based Photocatalytic Mechanisms

**Figure 10:**
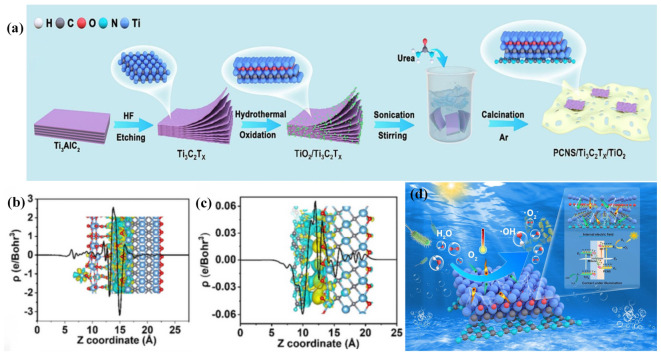
Structural design, interfacial charge redistribution, and photocatalytic antibacterial mechanism of PCNS/Ti_3_C_2_T_x_/TiO_2_ heterojunction photocatalysts. (**a**) Schematic illustration of the synthesis route for PCNS/Ti_3_C_2_T_x_/TiO_2_ heterojunction photocatalysts; (**b**) One-dimensional differential charge-density profile of the TiO_2_/Ti_3_C_2_O_2_ heterojunction; (**c**) One-dimensional differential charge-density profile of the PCNS/Ti_3_C_2_O_2_ heterojunction; (**d**) Proposed photocatalytic antibacterial mechanism of the PCNS/Ti_3_C_2_T_x_/TiO_2_ Z-scheme heterojunction under light irradiation. Reproduced from Ref. [130] with permission from Elsevier.

In MXene-assisted photocatalytic hydrogen evolution, CO_2_ reduction, and nitrogen reduction reactions, commonly referred to as HER, CO_2_RR, and NRR, macroscopic apparent quantum yields and ex situ characterizations alone are insufficient to reveal the intrinsic catalytic mechanisms [130]. Density functional theory (DFT) calculations therefore provide atomic-scale insights that complement experimental observations and help rationalize the structure–activity relationships of MXene-based photocatalysts (Figure 10).

DFT is particularly useful for understanding charge-transfer behavior in MXene-based heterojunctions. By calculating work functions, Fermi levels, and band alignments, DFT can estimate Schottky barrier heights, built-in electric fields, and the preferred migration direction of photogenerated carriers. This is especially important for MXenes because their metallic conductivity and Fermi-level positions are highly sensitive to surface terminations [34,131]. For instance, the Fermi level of Ti_3_C_2_T_x_ can be modulated over a broad range by varying the relative proportions of –F, –OH, and –O terminations [132,133], thereby influencing interfacial electron transfer towards MXene surfaces.

Beyond electronic structures, DFT critically maps the Gibbs free energy landscape of key photocatalytic surface reactions. For HER, an ideal cocatalyst should exhibit a hydrogen adsorption free energy, ΔGH, close to zero. However, pristine MXene surfaces often bind hydrogen too strongly, limiting their intrinsic activity [134]. Introducing oxygen terminations or constructing heterostructures with sulfides such as CdS can optimize ΔG_H and facilitate Volmer–Heyrovsky or Volmer–Tafel reaction pathways [135]. For CO_2_RR, DFT can identify how specific MXene surface sites stabilize key intermediates, such as •COOH, •CHO, and •CO, thereby regulating product selectivity toward CO, CH_4_, or HCOOH. Similarly, for NRR, calculations clarify N_2_ adsorption configurations (end-on vs. side-on) at edge or defect sites and identify strategies to lower the rate-determining step barrier while suppressing competing HER [136]. These insights illuminate how lowering the activation barrier of the rate-determining step not only facilitates nitrogen activation but also intrinsically suppresses the competitive HER. Future studies should integrate DFT with ab initio molecular dynamics, explicit solvent models, and machine learning-accelerated simulations to better bridge simplified theoretical models and real MXene-based photocatalytic systems.

### 3.5. Long-Term Stability and Deactivation Mechanisms of Mxene-Based Photocatalysts

**Figure 11:**
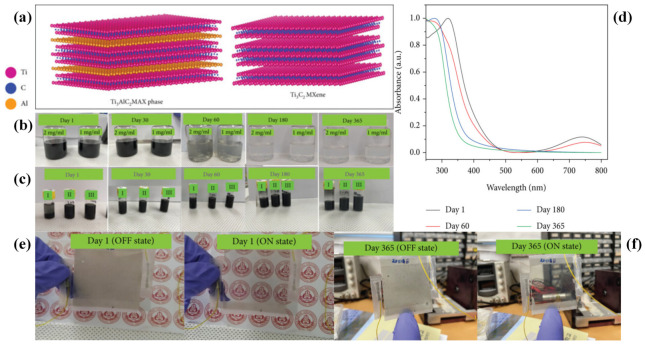
Long-term dispersion stability and functional retention of Ti_3_C_2_ MXene. (**a**) Structural comparison between the Ti_3_AlC_2_ MAX phase and Ti_3_C_2_ MXene phase; (**b**) Long-term dispersion stability of Ti_3_C_2_ MXene in deionized (DI) water at different concentrations; (**c**) Long-term dispersion behavior of Ti_3_C_2_ MXene in organic solvents: I, acetone; II, isopropyl alcohol (IPA); and III, ethanol; (**d**) Time-dependent UV–vis absorbance spectra of Ti_3_C_2_ MXene aqueous dispersions; (**e**,**f**) OFF/ON optical switching states of the PDLC MXene smart window on day 1 and day 365, respectively. Reproduced from Ref. [138] with permission from Wiley.

In photocatalytic energy conversion, long-term operational stability is a critical factor determining the practical applicability of catalysts. As shown in Figure 11a–d, the long-term dispersion behavior and time-dependent UV–vis absorbance of Ti_3_C_2_ MXene reveal its stability evolution in different storage media, which are closely related to the durability challenges of MXene-based photocatalysts. However, MXene materials are intrinsically susceptible to oxidation, especially in aqueous environments under continuous light illumination [137].

The deactivation of MXene-based photocatalysts is primarily driven by their thermodynamic instability. Dissolved oxygen, water molecules, and strongly oxidizing photogenerated holes can collectively attack Ti–C bonds within MXene nanosheets, leading to the collapse of the two-dimensional layered framework and the gradual conversion into TiO_2_-rich species and carbonaceous residues [138,139]. This self-oxidation, or photocorrosion, disrupts the metallic conductive network of MXenes, thereby weakening their function as electron sinks and damaging the interfacial contact within heterojunction systems [138,140]. In addition, prolonged photocatalytic operation may induce restacking or aggregation of MXene nanosheets, which further reduces exposed active sites and hinders interfacial charge transfer.

To address these stability limitations, several protection and passivation strategies have been proposed. One effective approach is interfacial engineering through Z-scheme heterojunction design. In such systems, strongly oxidizing holes are retained on the semiconductor side, such as TiO_2_ or graphitic carbon nitride, where they can be rapidly consumed by sacrificial reagents or oxidation reactions, while photogenerated electrons are transferred toward MXenes to drive reduction reactions [104,138,140]. This spatially separated charge-transfer pathway can effectively mitigate direct hole-induced oxidation of MXenes. Another important strategy is surface and edge capping. Because edge atoms in MXene nanosheets are coordinatively unsaturated and highly reactive, they often serve as initial sites for oxidative degradation [52]. Passivating these vulnerable sites with antioxidants, such as ascorbic acid or phytic acid, or with suitable polymer coatings can markedly enhance the colloidal and structural stability of MXenes in aqueous media. To advance this field toward practical application, future studies should prioritize the development of standardized accelerated aging protocols to rigorously quantify the photocorrosion kinetics, interfacial stability, and long-term performance retention of MXene-based photocatalysts under continuous illumination exceeding 1000 h.

Catalyst stability and recyclability are essential for evaluating the practical feasibility of MXene-based photocatalysts. Many studies report 3–5 cycling tests, but such short-term experiments are insufficient to demonstrate long-term durability under continuous operation. MXene nanosheets are susceptible to oxidation in aqueous and illuminated environments, leading to the formation of TiO_2_-rich species, loss of metallic conductivity, restacking, and deterioration of interfacial charge-transfer pathways. In addition, MXene-based composites containing CdS-, Cu_2_O-, and Ag-based compounds, Bi-based oxides, or Co or Ru cocatalysts may suffer from metal ion leaching or photocorrosion, which can cause secondary pollution and alter photocatalytic mechanisms. Therefore, future studies should combine cycling tests with post-reaction XRD, XPS, SEM/TEM, ICP–MS or ICP–OES leaching analysis, TOC removal, and toxicity assays to verify both catalytic durability and environmental safety.

### 3.6. Life Cycle Assessment and Techno-Economic Analysis

**Figure 12:**
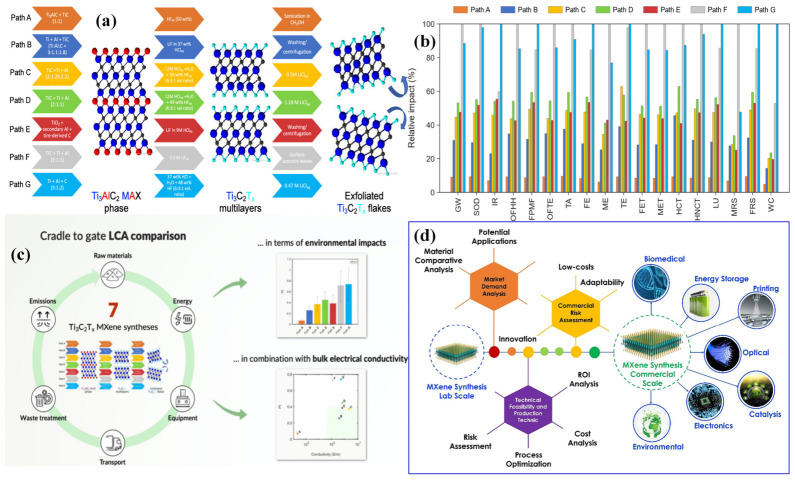
Life cycle assessment and techno-economic evaluation of Ti_3_C_2_T_x_ MXene. (**a**) Major stages involved in seven Ti_3_C_2_T_x_ MXene synthesis strategies evaluated by life cycle assessment (LCA); (**b**) Relative midpoint environmental impacts for producing 1 g of Ti_3_C_2_T_x_ MXene via different synthesis pathways, calculated using the modified ReCiPe 2016 hierarchical method; (**c**) Conceptual LCA framework for MXene-based photocatalysts. Reproduced from Ref. [141] with permission from the American Chemical Society; (**d**) Economic evaluation of MXene production from synthesis to commercialization. Reproduced from Ref. [144] with permission from the American Chemical Society.

The deployment of MXenes in sustainable energy production and environmental remediation requires a critical evaluation of whether the material life cycle itself is environmentally sustainable (Figure 12a–c). Life cycle assessment (LCA) provides an internationally standardized framework for quantifying the environmental footprint of photocatalytic technologies from raw material extraction to end-of-life treatment [141]. Conventional MXene synthesis generally involves high-temperature sintering of Ti_3_AlC_2_ MAX phases, followed by exfoliation and etching using concentrated hydrofluoric acid (HF) or LiF/HCl mixtures. These processes are energy intensive and rely on toxic and corrosive reagents, generating waste streams that require costly and complex treatment.

LCA studies have shown that producing MXenes via conventional HF-based etching can result in high global warming potential (GWP), human toxicity potential (HTP), and cumulative energy demand (CED), often exceeding those of conventional carbon- or silicon-based materials [142,143]. Therefore, emphasizing the carbon-free nature of photocatalytic hydrogen production while neglecting the environmental burden associated with MXene synthesis may lead to an incomplete sustainability assessment [144].

Techno-economic analysis (TEA) is closely linked to the development of greener and more scalable MXene synthesis pathways (Figure 12d) [144]. Recent progress has been made in fluoride-free etching strategies, including Lewis acid molten salt methods, electrochemical etching, and alkali-assisted hydrothermal approaches. Comparative LCA studies suggest that molten salt etching can avoid fluoride-associated ecotoxicity and enable the recovery or recycling of etching salts, thereby reducing the overall environmental footprint of MXene production. From an economic perspective, however, the production cost of laboratory-grade MXenes remains relatively high, making their use as disposable suspended photocatalysts in large-scale water treatment economically unfavorable [145].

Accordingly, practical commercialization of MXene-based photocatalysts may rely on two complementary strategies: (i) deploying MXenes exclusively as high-efficiency, ultra-low-loading cocatalysts (<5 wt%) to maximize the economic return per gram, and (ii) immobilizing these nanosheets within advanced membranes or macroscopic structural reactors to guarantee robust structural integrity across thousands of continuous catalytic cycles. Such a framework is essential for moving MXene-based photocatalysis beyond performance-driven material design toward practical, scalable, and environmentally responsible deployment.

## 4. Conclusions and Future Perspectives

This review summarizes recent progress in MXene-based photocatalysts for pharmaceutical wastewater remediation and sustainable energy conversion, including H_2_ evolution, CO_2_ reduction, and N_2_ reduction/fixation. The metallic conductivity, tunable surface terminations, rich interfacial chemistry, and heterojunction-forming capability of MXenes enable their use as electron mediators, cocatalysts, and structural components. These features facilitate charge separation, suppress electron–hole recombination, and promote surface redox kinetics in semiconductor-based photocatalytic systems.

Despite substantial progress, practical implementation remains limited by several key challenges. Scalable and environmentally benign synthesis routes are needed to replace hazardous HF-based etching methods. Such routes are also important for better controlling surface terminations and defect chemistry. In aqueous and oxidative environments, MXenes may undergo structural degradation, loss of conductivity, and heterojunction failure. This instability can compromise long-term photocatalytic performance.

Future research should move beyond empirical trial-and-error optimization toward mechanism-guided and data-driven materials design. Integrating DFT calculations, machine learning, high-throughput screening, and operando characterization can help identify suitable MXene compositions, surface terminations, active sites, and semiconductor partners, while clarifying charge-transfer pathways and deactivation mechanisms. Meanwhile, life cycle assessment and techno-economic analysis should be incorporated to evaluate environmental and economic feasibility. Particular attention should also be given to bifunctional photocatalytic platforms capable of simultaneously degrading pharmaceutical pollutants and generating renewable energy, such as photoreforming wastewater into H_2_ under ambient solar irradiation.

The future development of MXene-based photocatalysts should not focus solely on short-term activity. Instead, it should balance catalytic efficiency, stability, scalability, environmental footprint, and economic viability. Durability, reproducibility, environmental compatibility, and economic feasibility will determine whether MXene-based photocatalysis can move beyond laboratory-scale demonstrations. Addressing these challenges will be essential for advancing MXene-based photocatalytic systems toward practical and sustainable water–energy applications.

Several knowledge gaps remain in understanding pharmaceutical degradation over MXene-based photocatalysts under real environmental conditions. First, most mechanistic studies are conducted in single-pollutant model solutions, whereas real wastewater contains multiple pharmaceuticals, inorganic ions, natural organic matter, suspended solids, and microorganisms. The influence of these components on ROS generation, pollutant adsorption, and interfacial charge transfer is still poorly understood. Second, the relationship between dominant ROS and toxicity evolution remains unclear because radical trapping and LC–MS analyses alone cannot confirm detoxification. Third, the dynamic evolution of MXene surface terminations and oxidation state during photocatalysis is rarely monitored under operando conditions. Fourth, the roles of matrix-induced fouling, competitive adsorption, and catalyst passivation have not been quantified. Fifth, standardized protocols for comparing degradation kinetics, mineralization, catalyst stability, metal leaching, and ecotoxicity are lacking. Addressing these gaps requires operando spectroscopy, isotope labeling, high-resolution mass spectrometry, toxicity bioassays, realistic wastewater matrices, and continuous-flow validation.

## Figures and Tables

**Table 1:** Comparison of recent reviews on MXene-based photocatalysts and the novelty of the present review.

Reference	Scope of Review	Limitations	Supplement to This Review
Chen et al. [1]	Environmental Applications of MXene	There has been limited discussion on the coupling of photocatalysis and energy conversion in pharmaceutical wastewater.	This paper focuses on photocatalysis of pharmaceutical wastewater and its relationship to the conversion mechanisms of H_2_, CO_2_, and N_2_.
Solangi et al. [32]	General environmental pollutants	Focused mostly on dye removal; lacked depth in pharmaceutical pathways and energy conversion.	Bridges pharmaceutical remediation with solar-to-chemical energy conversion; provides structured mechanistic pathways for ROS.
Tawalbeh et al. [40]	Pharmaceutical compounds removal	Lacked detailed electronic structure insights and did not cover hydrogen or CO_2_ reduction.	Integrates DFT-calculated work functions of terminated MXenes with energy conversion (H_2_, CO_2_, N_2_).
Arshad et al. [44]	Adsorption & Photocatalysis of pharmaceuticals	Lacked critical evaluation of real-wastewater matrix effects, ecotoxicology, and reactor scaling.	Dedicated analysis of ecotoxicological evaluation and continuous photocatalytic membrane reactors.
Iravani and Varma [45]	MXene-based photocatalysts for the degradation of organic pollutants	Systemic shortcomings in energy conversion and interfacial engineering	This paper presents the conversion of H_2_, CO_2_, and N_2_ and discusses the structure–property relationship.
Xie and Zhang [46]	The Role of MXene in Photocatalysis	There has been limited discussion regarding pharmaceutical wastewater and toxicity assessment.	This paper integrates drug degradation, toxicity evolution, real water bodies, and engineering assessments.
This review	Remediation of Pharmaceutical Wastewater and Sustainable Energy Conversion	-	A Unified Explanation of Water Treatment and Energy Conversion Applications Based on Interfacial Charge Transfer Mechanisms

“-” indicates the default.

**Table 3:** Representative MXene-based photocatalysts for sustainable energy conversion.

Application	Catalyst	Role of MXene	Reaction Conditions	Main Products	Yield	Stability	References
H_2_ evolution	MXene/Zn_x_Cd_1−x_S	Electron extraction, suppression of recombination, and improvement of HER kinetics	Visible light	H_2_	14.17 mmol g^−1^ h^−1^	4 Cycles	[88]
H_2_ evolution	Ti_3_C_2_/CdIn_2_S_4_	Schottky junction, electron junction	Visible light	H_2_	9 μmol h^−1^; AQY 19.52% at 400 nm	Cycle stability	[118]
CO_2_ reduction	Ti_3_C_2_/g-C_3_N_4_/ZnO	Charge separation, CO_2_ activation	Light-driven CO_2_ reduction	CO, CH_4_	CO 6.41 μmol g^−1^ h^−1^; CH_4_ 0.26 μmol g^−1^ h^−1^	5 Cycles	[120]
CO_2_ reduction	TiO_2_/Ti_3_C_2_	Schottky junction	Light-driven CO_2_ reduction	CO, CH_4_	CO and CH_4_ are 2.8 and 4.0 times that of TiO_2_, respectively	Cycle stability	[121]
N_2_ fixation	RuO_2_/TiO_2_–Ti_3_C_2_	Electronic Transport Platforms and Catalysis	Xe lamp	N_2_/NH_4_^+^	425/4.37 μmol g^−1^ h^−1^	Cycle stability	[128]
N_2_ fixation	MXene/TiO_2_/Co	Co Sites Promote N_2_ Adsorption/Activation	UV–vis	NH_4_^+^	110 μmol g^−1^ h^−1^	Cycle stability	[129]

## Data Availability

No new data was used for the research described in the article.

## Abbreviations

The following abbreviations are used in this manuscript:

2D	Two-dimensional	LDH	Layered double hydroxide
4-CP	4-Chlorophenol	MMT	Montmorillonite
APAP	Acetaminophen	MQDs	MXene quantum dots
AQY	Apparent quantum yield	NRR	Nitrogen reduction reaction
CB	Conduction band	nZVI	Nanoscale zero-valent iron
CIP	Ciprofloxacin	rGO	Reduced graphene oxide
CO_2_	Carbon dioxide	ROS	Reactive oxygen species
DFT	Density functional theory	SMX	Sulfamethoxazole
HCl	Hydrochloride	SMZ	Sulfadimidine/Sulfamethazine
HER	Hydrogen evolution reaction	TC	Tetracycline
HTC	Hydrochlorothiazide	VB	Valence band
IBU	Ibuprofen	LCA	Life cycle assessment
CO_2_RR	Carbon dioxide reduction reaction	TEA	Techno-economic analysis
EPR	Electron paramagnetic resonance	PL	Photoluminescence
EIS	Electrochemical impedance spectroscopy		
